# Optimization of Feed Formulation, Feeding Rate, and Plant-Based Supplements for Efficient Rearing of the Superworm *Zophobas morio* (Fabricius) Under Tropical Conditions

**DOI:** 10.3390/insects17020138

**Published:** 2026-01-25

**Authors:** Jarongsak Pumnuan, Noratat Prachom, Somsak Kramchote

**Affiliations:** 1 School of Agricultural Technology, King Mongkut’s Institute of Technology Ladkrabang, Bangkok 10520, Thailand; 2 Department of Aquaculture, Faculty of Fisheries, Kasetsart University, Bangkok 10900, Thailand

**Keywords:** insect protein, protein innovation, feed optimization, plant supplement, growth performance

## Abstract

Insects are increasingly recognized as sustainable protein sources due to their high feed conversion efficiency and low environmental impact. Among them, the superworm, *Zophobas morio* (Fabricius) (Coleoptera: Tenebrionidae), has strong potential for large-scale production; however, optimized feeding strategies under tropical conditions remain limited. This study aimed (1) to determine the optimal feed formulations and feeding rate using wheat bran supplemented with the KMITL Protein Innovation source (a protein feed ingredient developed by the School of Agricultural Technology, King Mongkut’s Institute of Technology Ladkrabang, KMITL), and (2) evaluate the influence of plant-based supplementary foods on larval performance. In Phase I, larvae were reared on 13 formulations with three protein levels (CP00, CP21, and CP24) and five feeding rates (A–E). Diets CP21–21 and CP24–21 (21 and 24% CP; wheat bran/protein = 2:1) resulted in the highest survival (83.4–84.1%) and the lowest feed conversion ratios (FCR = 2.29–2.34). Moderate feeding rates (C–D; 925–1110 g feed per tray for 50 days) produced the greatest larval weights (700–760 mg), whereas ad libitum feeding provided no additional benefit. In Phase II, larvae reared on CP21–21 with a restricted rate of 1100 g per tray and supplemented with ten plant-derived foods achieved comparable final weights (716–760 mg), but survival varied significantly among treatments. Mulberry leaf yielded the highest survival (95.3%), followed by banana, watermelon rind, winter melon, and jicama (>90%). Pumpkin and jicama accelerated pupation and adult emergence, showing a female-biased sex ratio among emerged adults (59.2–65.5%), suggesting enhanced developmental rates. These results establish a practical framework for cost-effective and sustainable *Z. morio* production under tropical conditions, contributing to circular bioeconomy strategies and supporting insect-protein innovation.

## Simple Summary

Superworms (*Z. morio*) are attracting increasing interest as a sustainable alternative protein source; however, their growth performance strongly depends on feed quality. This study aimed to optimize feed formulation by supplementing wheat bran with a novel protein ingredient, KMITL Protein Innovation, and evaluate the additional benefits of selected plant-based supplements under tropical rearing conditions. Diets with different protein levels were tested to identify the most suitable formulation for larval growth and productivity. The results clearly showed that higher dietary protein significantly enhanced larval growth rate and final body weight, and reduced the rearing period to harvest compared to wheat bran alone. In addition, the inclusion of specific plant-derived supplements improved survival rate and promoted more uniform larval development. These findings confirm that both protein optimization and appropriate plant-based supplementation can markedly improve superworm production efficiency. This research provides practical feeding strategies for farmers to enhance productivity while potentially reducing production costs. Importantly, the study also supports the development of sustainable insect-based protein systems, contributing to food security and future nutrition.

## 1. Introduction

The accelerating growth of the global population, projected to exceed 9.7 billion by 2050, poses critical challenges to worldwide food and feed security [1]. Conventional livestock production systems require extensive land, water, and feed resources, contributing substantially to deforestation, biodiversity loss, and greenhouse gas emissions, as consistently documented across recent sustainability assessments [2,3,4]. Moreover, the rising costs and volatility of traditional protein sources, such as fishmeal and soybean meal, have intensified the search for sustainable alternatives to support aquaculture and livestock production. In this context, the Food and Agriculture Organization (FAO) has highlighted insects as a promising “future protein” due to their high feed conversion efficiency, low ecological footprint, and nutritional richness [1,3,5]. Globally, insect-based protein systems are now recognized as part of a circular bioeconomy model that can help close nutrient cycles, reduce organic waste, and enhance global food resilience amid climate and resource constraints.

Edible insects have gained significant attention as sustainable and nutritionally balanced protein sources for both human consumption and animal feed [2,5,6,7]. Recent reviews have further emphasized that edible insects offer nutrient-dense biomass with sustainability benefits, supporting diverse applications across modern food and feed systems [7,8]. More than 2000 species of insects are consumed globally, many of which offer high protein levels (40–75%), essential amino acids, vitamins, and minerals comparable to or exceeding those of conventional meat and fish [5,6]. Insect rearing requires far less land, water, and feed input, and produces substantially lower greenhouse gas emissions than livestock systems [3,9]. Furthermore, insects can be reared on agricultural by-products, thereby transforming organic waste into valuable biomass and contributing to circular economy principles [10]. Numerous studies have confirmed that insect meals can partially or fully replace fishmeal and soybean meal in poultry, pig, and aquaculture feeds without compromising growth or feed conversion efficiency [2,8]. Consequently, insects represent not only an ecological alternative but also a strategic solution for strengthening global food security and sustainable protein supply chains.

Among various edible insects, the superworm, *Z*. *morio* (Coleoptera: Tenebrionidae) has gained attention for its rapid growth, high biomass yield, and nutrient density [11]. Recent studies have reported that *Z. morio* larvae typically attain 640–760 mg within 60–75 days under controlled laboratory or semi-industrial conditions, showing efficient feed utilization and high survival rates [12,13]. The pupal stage generally lasts about 13–15 days, followed by adult emergence that can persist for several months, allowing multiple reproductive cycles and efficient colony maintenance [11]. Compared with *Tenebrio molitor*, *Z. morio* exhibits higher feed intake, nutrient assimilation efficiency, and productivity [11,14]. These advantages are linked to its larger body size, efficient utilization of fibrous substrates, and faster conversion of feed into biomass, resulting in superior growth and yield under rearing conditions [11,12]. Recent studies further emphasize that the productivity and bioconversion efficiency of *Z. morio* are strongly influenced by rearing system design and diet optimization under commercial-scale conditions. Advances in intensive and semi-industrial rearing have demonstrated improved survival, feed utilization, and system stability, supporting the role of *Z. morio* as a scalable insect-protein species within circular bioeconomy frameworks [15,16]. The nutrient profile of *Z. morio* includes 39–46% CP and 30–36% lipids (dry weight), rich in lysine, methionine, and unsaturated fatty acids, making it a strong alternative to fishmeal and soybean meal [6,14]. The 6–8% chitin fraction provides antimicrobial and immunomodulatory benefits [17]. Biologically, *Z. morio* tolerates 25–32 °C and 55–75% humidity, thriving on low-cost agro-industrial by-products such as wheat bran and vegetable residues [5,10]. With low mortality, high pupation success, and efficient feed conversion [9], *Z. morio* serves as a bioconversion agent transforming agricultural waste into sustainable protein. These features align with the FAO’s [1] framework for circular bioeconomy and global food security, highlighting *Z. morio* as a key future protein resource.

The rearing efficiency of *Z. morio* is primarily influenced by diet formulation, feeding quantity, and environmental factors such as temperature, humidity, and population density. Previous studies have shown that this species thrives under semi-industrial rearing systems, with optimal conditions of 25–32 °C and 55–75% relative humidity [11]. Wheat bran has been widely accepted as a cost-effective basal diet because of its balanced nutrient profile and high palatability [5]. Nevertheless, feed optimization—primarily through the inclusion of locally available agro-industrial by-products such as cassava meal, rice bran, vegetable trimmings, or fruit residues—remains essential to enhance larval growth, feed conversion efficiency, and survival while minimizing rearing costs [1,10]. Recent research has shown that dietary supplementation using agro-industrial by-products or plant-derived functional ingredients can significantly enhance larval performance in *Z. morio*, improving growth rate, feed conversion efficiency, and survival [12,18]. In response to these limitations, the KMITL Protein Innovation feed ingredient, developed by the School of Agricultural Technology, King Mongkut’s Institute of Technology Ladkrabang (KMITL), is introduced as a proprietary protein source formulated with balanced amino acids and energy to support biomass growth in *Z. morio*. The KMITL Protein Innovation feed ingredient is a soy protein-based supplement formulated with reprocessed aquafeed materials and a vitamin–mineral premix to provide a balanced amino profile and support efficient growth and feed utilization [19]. It is available in multiple protein levels and is recommended for use alongside wheat bran—the primary carbohydrate base in superworm rearing—to allow flexible protein adjustment under different rearing conditions. However, evidence regarding the optimal type and inclusion level of such supplementary feeds under tropical rearing systems remains limited. Therefore, a systematic evaluation of plant-based supplements is essential to determine cost-effective formulations that promote growth efficiency and survival, while supporting sustainable insect-protein production in tropical environments.

Although *Z. morio* has gained attention as a sustainable protein source, studies on feed optimization and the efficient utilization of locally available plant-based ingredients remain limited. Most research has relied on wheat bran-based diets, which may not suit tropical rearing systems in Southeast Asia. Therefore, this study aimed (i) to develop an optimized feed formulation and feeding rate using wheat bran combined with the KMITL Protein Innovation source, and (ii) to evaluate the effects of plant-derived supplementary foods on larval growth, survival, and adult emergence. The findings are expected to provide a scientific basis for developing cost-effective and sustainable feeding strategies under tropical bioeconomy conditions.

## 2. Materials and Methods

### 2.1. Insect Culture and Feed Formulation

A stock culture of the superworm *Z. morio* was established using adult beetles obtained from a commercial insect farm in Nong Chok district, Bangkok, Thailand. Adults were maintained in plastic trays (28 × 42 × 9.5 cm) lined with clean white paper and filled with wheat bran, which served as both the oviposition substrate and the initial food source. After oviposition, egg-containing paper sheets were carefully transferred to new trays and supplemented with wheat bran as a basal diet. Larvae emerging from these eggs were designated as newly hatched larvae and reared under natural light and ambient (non-air-conditioned) laboratory conditions in Bangkok, Thailand, with temperature and relative humidity monitored and typically averaging 28–34 °C and 70–80%, respectively. To maintain adequate moisture, fresh jicama slices (100 g) were provided at each feeding, and distilled water was lightly sprayed onto the substrate twice daily (09:00 and 16:00). From this cohort, 20-day-old larvae (mean body weight of 75.3 ± 11.3 mg), determined from a random subsample of 50 larvae prior to the experiment were selected as starter insects for subsequent feed formulation trail.

### 2.2. Feed Formulation and Feeding Rate Effects on Superworm Growth

#### 2.2.1. Screening of Feed Formulations

This phase aimed to identify the most suitable feed formulations for efficient superworm rearing. The experiment was adapted and modified from Lomwong et al. [20]. Thirteen feed formulations were prepared using wheat bran as the basal ingredient and the KMITL Protein Innovation source as the protein supplement. The formulations included CP00–10 (wheat bran only, serving as the control); CP18–01, –11, –12, and –21, containing 18% protein with wheat bran-to-protein ratios of 0:1, 1:1, 1:2, and 2:1, respectively; CP21–01, –11, –12, and –21, containing 21% protein with the same ratios; and CP24–01, –11, –12, and –21, containing 24% protein with the same ratios. The dietary crude protein (CP) levels used in this study (18, 21, and 24%) were selected based on two considerations: (i) wheat bran, widely used as a low-cost basal feed ingredient, typically contains approximately 14–17% CP; and (ii) CP levels of approximately 20–35% are commonly reported for herbivorous and omnivorous animals, depending on species and growth stage [21,22]. Accordingly, the selected CP range enables evaluation of protein supplementation effects above the basal wheat bran level under biologically relevant and economically realistic rearing conditions.

All ingredients were ground to a uniform consistency, thoroughly mixed to ensure homogeneity, and stored in airtight containers at room temperature until use. A completely randomized design (CRD) with five independent biological replicates was employed. Prior to the screening experiment, preliminary trials were conducted to stabilize rearing conditions and refine feeding protocols. Each replicate consisted of 100 g of 20-day-old larvae reared in a plastic tray under controlled laboratory conditions. The initial larval number was estimated from the total biomass and mean individual body weight (approximately 1300 larvae per replicate). Survival rate was calculated based on the estimated initial larval number and the final number of live larvae counted at the end of the experiment. Larvae were fed ad libitum and supplemented with 100 g of fresh jicama slices as a moisture source. Water was sprayed onto the substrate twice daily (09:00 and 16:00 h) to prevent desiccation. Feed was replaced every five days, and the rearing period lasted 70 days. Survival rate, body weight, and feed conversion ratio (FCR) were recorded. Survival rate (%) was calculated as the ratio of surviving larvae to the initial number introduced. Average body weight (mg) was determined at the end of the 70-day rearing period from a subsample of 50 larvae per replicate. The FCR was calculated as *F*/(*W*_*f*_ − *W*_*i*_), where *F* represents the cumulative feed input per replicate, and *W_f_* and *W_i_* denote the final and initial total larval biomass, respectively, following standard approaches used in edible insect studies [11,12].

#### 2.2.2. Determination of Optimal Feeding Quantity

Based on the results of the screening phase, the CP21–21 and CP24–21 KMITL feed formulations, which previously showed high survival rates and favorable FCRs, were selected for this experiment, along with the basal diet CP00–10 as a control. The experiment was conducted using a CRD with five biological replicates per treatment. This phase was designed as a confirmatory trial based on optimized rearing conditions established during the preliminary experiments. Each replicate consisted of 100 g of 20-day-old larvae reared in a plastic tray. Feeding was carried out at 5-day intervals following the progressive schedule shown in Table 1, in which feed quantity increased with larval age. The total feed amounts ranged from 555 to 1850 g per tray (equivalent to ≈5.6–18.5 g feed g^−1^ initial biomass over the 50-day rearing period). These feeding levels were established through preliminary trials ranging from restricted feeding to practical satiation (A to D), with level E representing an upper-bound reference for near ad libitum feeding under tray-based conditions rather than a target for fine-scale optimization. Accordingly, feeding rate was expressed on a per-tray basis and normalized to initial larval biomass, as the experimental unit was the tray-based biomass. This approach reflects practical production conditions and ensures comparability among treatments. Fresh jicama slices (100 g) were added at each feeding to maintain moisture, and distilled water was lightly sprayed twice daily (09:00 and 16:00 h). Larvae were randomly sampled (*n* = 50 individuals per replicate) and weighed at 5-day intervals until 70 days of age. The 70-day trial was terminated when most larvae reached the prepupal stage, indicated by cessation of feeding, reduced mobility, and characteristic body contraction prior to pupation. Response variables included larval body weight, survival rate, and feed conversion ratio (FCR). Recorded parameters included larval body weight, survival rate, and FCR.

### 2.3. Supplementary Food Effects on Superworm Growth

This experiment was conducted to evaluate the effects of different supplementary foods on the larval growth, adult emergence, and sex ratio of *Z. morio*. All treatments were reared on the CP21–21 KMITL feed formulation, consisting of wheat bran combined with the 21% protein formulation at a 2:1 ratio, which had previously been identified as the most suitable basal diet in the preceding experiment. The total feed quantity was fixed at 1100 g per tray, corresponding to feeding level D, the optimal feeding quantity determined in the previous phase.

Nine types of supplementary foods were tested, including jicama (*Pachyrhizus erosus*), pumpkin (*Cucurbita moschata*), banana (*Musa* (ABB group) ‘Kluai Namwa’), cucumber (*Cucumis sativus*), watermelon peel (*Citrullus lanatus*), winter melon (*Benincasa hispida*), mulberry leaf (*Morus alba*), carrot (*Daucus carota*), Chinese cabbage (*Brassica rapa* subsp. *chinensis*), and a non-supplemented control, in which larvae were provided with the basal CP21–21 diet only, without any additional plant-based supplementary food. Supplementary foods were supplied ad libitum (≈100 g per feeding) and replenished as needed depending on larval consumption. Feeding frequency was adjusted according to larval age, being less frequent during early instars and more frequent as larvae matured.

At 70 days of age, superworms were randomly sampled for sex ratio analysis, with 50 larvae selected per replicate from each treatment (three biological replicates per treatment) and individually transferred into 250 mL clear glass cups without lids. Each larva was reared singly with wheat bran and supplemented with the same type of food as in the previous feeding phase until pupation and adult emergence. The survival rate during pupation, specific growth rate (SGR, %), and the proportions of the sexes among emerged adults were subsequently recorded. The SGR was calculated as [(ln *W*_*f*_ − ln *W*_*i*_) × 100]/*t*, where *W_f_* and *W_i_* represent the final and initial larval body weights, respectively, and *t* is the rearing duration (days), following the general approach used in edible insect studies [11,12].

### 2.4. Data Analysis

All data on larval body weight, survival rate, feed conversion ratio (FCR), specific growth rate (SGR), adult emergence, and sex ratio were analyzed using analysis of variance (ANOVA) under a completely randomized design (CRD). The number of biological replicates depended on the experimental phase, with five replicates used for feeding quantity and growth performance experiments, and three biological replicates used for sex ratio analysis. Mean separations were performed using Duncan’s Multiple Range Test (DMRT) at a significance level of *p* < 0.01. Statistical analyses were conducted using SPSS software, version 16.0 (IBM Corp., Armonk, NY, USA).

## 3. Results

### 3.1. Feed Formulation and Feeding Quantity Effects on Superworm Growth

Larval survival and feed utilization of *Z. morio* were significantly influenced by feed formulation and protein composition (Table 2). Among all treatments, the CP24–21 formulation yielded the highest larval survival (84.1%), followed closely by CP21–21 (83.4%) and CP00–10 (83.2%), whereas CP21–01 showed the lowest survival (65.2%). Feed conversion ratio (FCR) values ranged from 2.29 to 5.20, with the lowest FCR values recorded in CP24–21 (2.29) and CP21–21 (2.34). These results show that feed formulations containing 21–24% CP, when combined with wheat bran at a 2:1 ratio, were associated with higher larval survival and lower FCR values under controlled laboratory conditions (*p* < 0.01). Based on the observed survival rates and feed conversion ratios, CP21–21 and CP24–21 were selected as candidate feed formulations for further evaluation in subsequent experiments.

**Figure 1:**
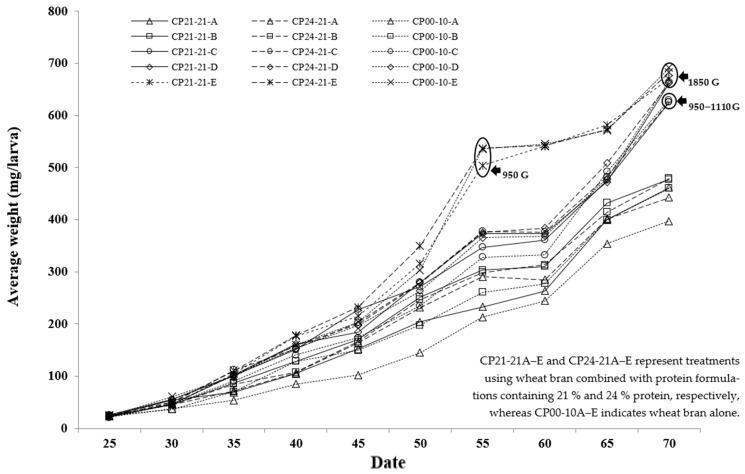
Average body weight (mg per larva) of superworms (*Z. morio*) at different ages when reared on KMITL feed formulations (CP00, CP21, and CP24) from 20 to 70 days of age. Values represent mean body weight (*n =* 50 larvae per replicate, five replicates per treatment).

Larval body weight of *Z. morio* increased continuously from 20 to 70 days across all treatments (Figure 1). Although larvae under ad libitum feeding (level E) exhibited rapid growth during the early rearing phase, their final body weights at 70 days were comparable to those reared at level D but significantly higher than those at level C. This suggests that excessive feeding did not further enhance growth performance under controlled conditions. At 70 days, larvae fed with the CP21–21 and CP24–21 formulations achieved the highest final weights (537–759 mg) and superior growth performance compared with the control (CP00–10) (Table 3). The most efficient feed conversion ratios (FCR = 1.43–1.44) were recorded at feeding level C, while survival rates remained high (77–85%) across all treatments. Overall, these findings indicate that moderate feeding level D (≈1110 g per tray) provided optimal nutrient utilization and larval growth efficiency without unnecessary feed loss. Therefore, CP21–21 and CP24–21 at level D can be recommended as the most effective feeding strategies for *Z. morio* rearing under controlled laboratory conditions.

### 3.2. Supplementary Food Effects on Superworm Growth

**Figure 2:**
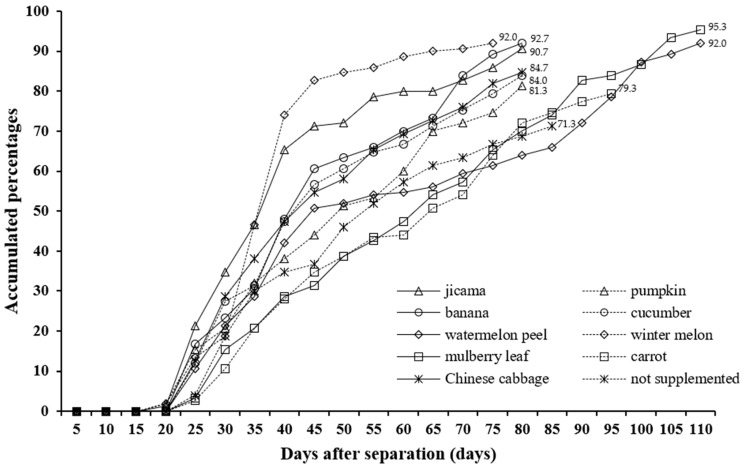
Accumulated percentages of superworms (*Z. morio*) that developed into adults and the survival of emerged adults when reared individually (single rearing) after 70 days of age and fed with various supplementary foods.

**Figure 3:**
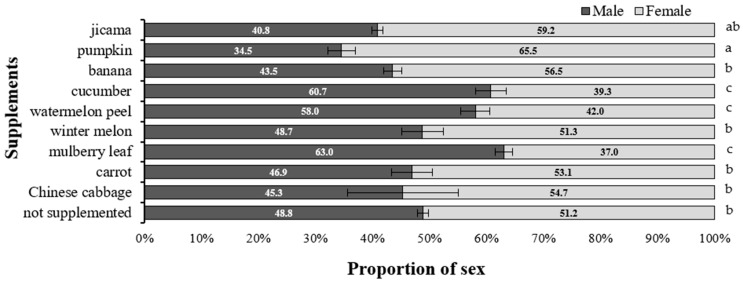
Proportion of male and female adult superworms (*Z. morio*) reared on the CP21–21 KMITL feed formulation (1100 g) with various food supplements. Means followed by different letters indicate significant differences (*p* < 0.01, DMRT).

Larval growth, survival, and developmental performance of *Z. morio* were evaluated under a limited feeding regime of 1100 g per tray, corresponding to the D feeding level determined in the previous experiment. As shown in Table 4, the addition of various supplementary foods did not significantly affect final larval mass after 70 days, with body weights ranging from 716.4 to 760.0 mg and specific growth rates (%SGR) ranging from 4.48 to 4.61% per day, showing no significant differences among treatments. However, survival rates varied markedly among treatments, with the highest rate (95.3%) observed in larvae fed mulberry leaves, followed by those fed banana, watermelon peel, and winter melon (over 90%), whereas the lowest rate was observed in the non-supplemented control (71.3%). The cumulative adult emergence patterns (Figure 2) showed that larvae fed pumpkin, banana, or mulberry leaves pupated and emerged earlier than those fed other treatments, indicating faster development under these diets. In contrast, the control and Chinese cabbage groups exhibited slower pupation and lower emergence rates. Sex ratio data (Figure 3) indicated that adult sex composition differed among supplement treatments. Female proportions were highest in the pumpkin treatment (65.5%), followed by jicama and banana, whereas cucumber, mulberry leaf, and watermelon peel produced male-biased ratios (Figure 3). Under the moderate feeding level (D; 1100 g tray^−1^), supplemental foods did not significantly affect larval body weight or specific growth rate; however, treatments differed in survival, the timing of pupation and adult emergence, and adult sex composition.

## 4. Discussions

### 4.1. Effects of Feed Formulation and Feeding Quantity on Superworm Growth

The results clearly demonstrated that feed formulations containing 21–24% CP, mixed with wheat bran at a 2:1 ratio, yielded the highest survival rates and the most efficient feed conversion in *Z. morio*. This outcome reinforces the importance of balanced macronutrient ratios, where optimal growth in Tenebrionidae larvae depends not only on protein concentration but also on the protein–carbohydrate energy balance [23]. A similar trend was also observed in *Tenebrio molitor* by Syahrulawal et al. [24], who showed that larval growth and nutritional quality are optimized when protein and carbohydrate levels are balanced and the feed matrix remains highly digestible. The superior performance of our bran-based diets may be attributed to both nutrient synergy and gut physiological compatibility. Wheat bran provides digestible carbohydrates, dietary fiber, and essential minerals that stimulate microbial fermentation and nutrient assimilation [11,25]. When combined with moderate protein enrichment from the KMITL Protein Innovation source, the 2:1 ratio likely achieved an optimal nutrient balance similar to that observed in *T. molitor* and *Z. morio* when the dietary protein-to-carbohydrate ratio ranged from 1.5:1 to 2:1 [12]. Dragojlović et al. [14] further showed that such balance supports superior biochemical composition—including elevated lysine and unsaturated fatty acids—highlighting the nutritional relevance of this formulation.

Our study also revealed that moderate feeding levels (C–D; approximately 925–1110 g per tray) produced larval body weights and survival rates comparable to those under ad libitum feeding, but with markedly lower feed conversion ratios. This pattern indicates that excess feeding beyond nutritional requirements offers no growth advantage and may instead reduce feed efficiency—a phenomenon observed across Tenebrionidae larvae [23,26]. Overfeeding increases moisture and microbial load in rearing trays, deteriorating substrate quality and consequently slowing larval metabolism and nutrient assimilation [24]. Overfeeding likely increased substrate moisture and microbial load, reducing feed conversion efficiency as also reported by Harsányi et al. [27]. Therefore, moderate feeding regimes are more practical under tropical rearing systems. Thus, our evidence supports the adoption of controlled, moderate feeding regimes as a practical management approach for tropical insect farming systems.

Such optimization is particularly critical under tropical rearing conditions, where temperature (28–34 °C) and humidity (70–80%) can accelerate feed spoilage. By moderating feeding rates, producers can minimize microbial proliferation, stabilize humidity within rearing trays, and extend the usable life of the substrate. The combination of a balanced feed formulation (21–24% CP, 2:1 ratio) and moderate feed provision (C–D levels) thus represents a scientifically validated, cost-effective, and sustainable strategy for large-scale *Z. morio* production. From a practical perspective, these optimized feeding and management practices may translate into tangible farm-level benefits for insect farmers, particularly in terms of income generation and food security, as insect farming has been recognized as a viable livelihood option under resource-limited conditions [4]. Improved production efficiency and reduced operational risks may support more stable yields, thereby contributing to household welfare and food security [28]. Importantly, the realization of these benefits depends on farmers’ perceptions and access to appropriate information, which strongly influence technology adoption and productivity [29,30]. In the longer term, system-level integration of emerging agricultural technologies may further enhance these outcomes by improving resource-use efficiency and production stability [31].

### 4.2. Supplementary Food Effects on Superworm Growth

Under a fixed moderate feeding regime, the type of supplementary food clearly affected larval performance. Supplements such as mulberry leaf, banana, and pumpkin increased survival and accelerated emergence. However, final body mass and %SGR (≈4.48–4.61% day^−1^) remained statistically similar among treatments (Table 4; Figure 2 and Figure 3). These results suggest that the CP21–21 basal formulation already provided adequate protein and energy. Plant supplements, therefore, acted mainly as physiological stabilizers. They supplied moisture, soluble carbohydrates, fiber, and micronutrients that supported gut function and reduced desiccation stress under tropical conditions—this improved feed-to-biomass conversion without increasing final weight. Comparable findings were reported by Gourgouta et al. [12], who observed that plant-residue diets enhanced *Z. morio* survival without affecting mass. Likewise, Liu et al. [26] showed that fresh plant materials improved *T. molitor* growth through better digestibility and water balance. More recent trials also confirmed that wheat-based substrates enriched with plant by-products maintained high growth and feed efficiency in Tenebrionidae larvae [25,32,33]. Collectively, these studies support our interpretation that functional plant supplements improve nutrient assimilation and microclimatic stability rather than macronutrient supply per se.

Across treatments, adult sex composition differed among supplementary food types (Figure 3). Female proportions exceeded 50% in several treatments, with the highest female share observed under pumpkin supplementation, followed by jicama and banana, whereas cucumber, mulberry leaf, and watermelon peel exhibited male-biased sex ratios. Because sex determination in Tenebrionidae is genetically fixed, these shifts likely reflect sex-specific differences in survival and developmental success during the larval and pupal stages rather than changes in primary sex determination. Similar diet patterns have been reported previously in *Z*. *morio* colonies under varying dietary and rearing conditions. Management factors, including dietary quality and rearing environment, are known to influence adult sex ratios through differential survival. A balanced or slightly female-biased sex ratio is generally considered favorable for productivity in mass-rearing systems [34]. Evidence from mealworm production likewise indicates that husbandry variables, such as substrate type and breeding density, can modulate reproductive performance and cohort sex composition, underscoring the importance of monitoring sex ratios alongside survival parameters when evaluating dietary effects [11,35]. In the present study, female-skewed ratios observed under pumpkin- and banana-based supplementation are therefore consistent with reported associations between diet quality and sex-specific survival during development, although the underlying physiological mechanisms were not directly examined. Because sex ratios were assessed only among successfully emerged adults, the observed patterns should be interpreted as outcomes of differential survival rather than direct measures of sex determination.

From a practical production perspective, the lack of differences in final body weight and specific growth rate among treatments indicates that plant-based supplementary foods primarily improve survival rather than enhance individual growth. In this study, several locally available supplements increased survival by approximately 10–20% compared with the non-supplemented control, resulting in a higher number of harvestable individuals despite unchanged individual biomass. Such survival gains are particularly relevant for small- to medium-scale insect farming systems, where total output is strongly influenced by cohort survival. In addition, tendencies toward more balanced or female-skewed sex ratios under certain supplements may offer further advantages in breeding-oriented systems. Given that many plant-based supplements can be sourced locally as low-value by-products, these survival benefits may offset additional feed costs, supporting cost-efficient management decisions at the farm level [4,29].

## 5. Conclusions

This study identified an efficient feeding strategy for rearing the superworm, *Z. morio* under tropical conditions. The CP21–21 formulation, with a moderate feeding rate (≈1100 g per tray, equivalent to 11 g feed g^−1^ initial biomass), provided a balance among nutrient utilization, larval survival, and growth performance. Supplementation with moisture-rich plant materials such as pumpkin, banana, and mulberry leaf further improved survival and pupation efficiency without prolonging development. These plant supplements are locally available and low-cost; their use may enhance the economic feasibility and sustainability of superworm production in tropical regions.

## Figures and Tables

**Table 1:** Feeding schedules and total food quantities provided to superworm *Z. morio* larvae at 5-day intervals during the 20–70-day rearing period.

Food Formulas	The Amount of Food (g) per Tray											Total (g)
	20	25	30	35	40	45	50	55	60	65	70	
A	15	30	30	30	60	60	60	90	90	90	-	555
B	20	40	40	40	80	80	80	120	120	120	-	740
C	25	50	50	50	100	100	100	150	150	150	-	925
D	30	60	60	60	120	120	120	180	180	180	-	1110
E	50	100	100	100	200	200	200	300	300	300	-	1850

A to E correspond to different feeding treatments, with total feeding quantities of 555, 740, 925, 1110, and 1850 g per tray, respectively (equivalent to 5.6–18.5 g feed g^−1^ initial biomass over the 50-day period). Each feeding level was applied across all feed formulations (CP00–10, CP18–01 to CP24–21).

**Table 2:** Survival rate and feed conversion ratio (FCR) of 70-day-old superworms (*Z. morio*) reared on different feed formulations from 20 to 70 days of age.

KMITL		Mean ^1^ ± SD	
Feed Formulation ^2^	Survival Rate (%)		FCR ^3^
CP00–10	83.2 ± 3.8 ^ab^		2.56 ± 0.18 ^cd^
CP18–01	72.2 ± 7.0 ^cd^		5.20 ± 0.20 ^a^
CP18–11	75.1 ± 6.2 ^bc^		2.71 ± 0.28 ^c^
CP18–12	80.9 ± 3.6 ^ab^		2.60 ± 0.26 ^cd^
CP18–21	80.6 ± 1.7 ^abc^		2.56 ± 0.36 ^cd^
CP21–01	65.2 ± 9.9 ^d^		4.64 ± 0.25 ^b^
CP21–11	77.3 ± 4.1 ^abc^		2.62 ± 0.17 ^cd^
CP21–12	80.8 ± 2.7 ^abc^		2.43 ± 0.21 ^cd^
CP21–21	83.4 ± 6.0 ^ab^		2.34 ± 0.20 ^cd^
CP24–01	67.0 ± 8.8 ^d^		4.65 ± 0.30 ^b^
CP24–11	76.3 ± 6.6 ^abc^		2.60 ± 0.27 ^cd^
CP24–12	77.5 ± 6.3 ^abc^		2.60 ± 0.30 ^cd^
CP24–21	84.1 ± 3.8 ^a^		2.29 ±0.24 ^d^

^1^ Means within the same column followed by the same letter are not significantly different (*p* < 0.01) according to Duncan’s Multiple Range Test (DMRT). ^2^ CP00–10 = wheat bran alone (control); CP18–01 to CP18–21, CP21–01 to CP21–21, and CP24–01 to CP24–21 = wheat bran mixed with KMITL Protein Innovation formulations containing 18%, 21%, and 24% protein, respectively, at ratios of 0:1, 1:1, 1:2, and 2:1. ^3^ FCR = feed conversion ratio.

**Table 3:** Average body weight, feed conversion ratio (FCR), and survival percentage of superworms (*Z. morio*) aged 70 days reared on different KMITL feed formulations (CP00, CP21, and CP24) from 20 to 70 days of age.

KMITL Feed Formulation ^2^	Feeding Levels	Mean ^1^ ± SD		
		Body Weight (mg/Larva)	FCR ^3^	Survival Percentages
CP21–21	A	537.3 ± 16.4 ^cd^	1.16 ± 0.44 ^g^	77.4 ± 7.1 ^a^
	B	553.4 ± 17.6 ^c^	1.49 ± 0.43 ^de^	81.0 ± 6.5 ^a^
	C	701.3 ± 25.1 ^b^	1.44 ± 0.31 ^e^	74.9 ± 4.9 ^a^
	D	738.4 ± 22.2 ^a^	1.63 ± 0.27 ^b^	82.9 ± 4.3 ^a^
	E	747.4 ± 12.2 ^a^	2.58 ± 0.12 ^a^	85.0 ± 3.1 ^a^
CP24–21	A	517.9 ± 24.8 ^d^	1.21 ± 0.50 ^g^	77.0 ± 9.6 ^a^
	B	555.1 ± 13.4 ^c^	1.49 ± 0.35 ^de^	80.0 ± 9.1 ^a^
	C	702.2 ± 16.3 ^b^	1.43 ± 0.30 ^e^	75.2 ± 2.6 ^a^
	D	738.9 ± 9.6 ^a^	1.63 ± 0.19 ^b^	81.5 ± 3.4 ^a^
	E	759.0 ± 12.3 ^a^	2.56 ± 0.04 ^a^	83.4 ± 2.3 ^a^
CP00–10	A	473.5 ± 17.8 ^e^	1.33 ± 0.40 ^f^	79.5 ± 9.3 ^a^
	B	536.3 ± 22.1 ^cd^	1.55 ± 0.38 ^cd^	80.3 ± 5.1 ^a^
	C	706.5 ± 27.3 ^b^	1.43 ± 0.25 ^e^	75.6 ± 2.9 ^a^
	D	754.8 ± 45.2 ^a^	1.59 ± 0.22 ^bc^	81.0 ± 3.8 ^a^
	E	767.9 ± 20.1 ^a^	2.60 ± 0.07 ^a^	82.4 ± 3.6 ^a^

^1^ Means within the same column followed by the same letter are not significantly different (*p* < 0.01) according to DMRT. ^2^ CP00–10 = wheat bran only; CP18–01 to CP18–21 = wheat bran: P18 (KMITL formulation, 18% protein) = 0:1, 1:1, 1:2, and 2:1; CP21–01 to CP21–21 = wheat bran: P21 (KMITL formulation, 21% protein) = 0:1, 1:1, 1:2, and 2:1; CP24–01 to CP24–21 = wheat bran: P24 (KMITL formulation, 24% protein) = 0:1, 1:1, 1:2, and 2:1. Feeding levels A–E indicate limited food quantities of 555, 740, 925, 1110, and 1850 g per tray, respectively. ^3^ FCR = feed conversion ratio.

**Table 4:** Average body weight, specific growth rate (%SGR), and survival percentage of 70-day-old superworms (*Z. morio*) reared on the CP21–21 KMITL feed formulation under a limited feeding quantity of 1100 g with various supplementary foods from 20 to 70 days of age.

Food Supplements	Mean ^1^ ± SD		
	Body Weight (mg/Larva)	Survival Percentages	%SGR
jicama	746.4 ± 95.8 ^a^	90.7 ± 2.1 ^b^	4.57 ± 0.25 ^a^
pumpkin	748.4 ± 113.6 ^a^	81.3 ± 1.0 ^cd^	4.57 ± 0.30 ^a^
banana	760.0 ± 101.9 ^a^	92.7 ± 1.8 ^ab^	4.61 ± 0.27 ^a^
cucumber	758.6 ± 111.8 ^a^	84.0 ± 1.8 ^c^	4.60 ± 0.31 ^a^
watermelon peel	730.2 ± 85.8 ^a^	92.0 ± 1.8 ^ab^	4.53 ± 0.23 ^a^
winter melon	755.0 ± 106.2 ^a^	92.0 ± 3.6 ^ab^	4.59 ± 0.28 ^a^
mulberry leaf	742.0 ± 109.8 ^a^	95.3 ± 2.7 ^a^	4.55 ± 0.31 ^a^
carrot	738.2 ± 110.0 ^a^	79.3 ± 2.7 ^d^	4.54 ± 0.29 ^a^
Chinese cabbage	757.6 ± 82.2 ^a^	84.7 ± 1.0 ^c^	4.61 ± 0.23 ^a^
not supplemented	716.4 ± 108.6 ^a^	71.3 ± 1.0 ^e^	4.48 ± 0.30 ^a^

^1^ Means within the same column followed by the same letter are not significantly different (*p* < 0.01) according to DMRT. All treatments were reared on the CP21–21 KMITL feed formulation (21% protein) under a limited feeding quantity of 1100 g.

## Data Availability

The raw data supporting the conclusions of this article will be made available by the authors on request.
